# Cervical Human Papillomavirus Screening among Older Women

**DOI:** 10.3201/eid1111.050575

**Published:** 2005-11

**Authors:** Matthew J. Grainge, Rashmi Seth, Li Guo, Keith R. Neal, Carol Coupland, Paul Vryenhoef, Jane Johnson, David Jenkins

**Affiliations:** * University of Nottingham Medical School, Nottingham, United Kingdom; † Nottingham City Hospital, Nottingham, United Kingdom

**Keywords:** human papillomavirus, cervix neoplasms, incidence, natural history, age factors, epidemiologic studies, retrospective studies, research

## Abstract

Rates of acquisition and clearance of cervical human papillomavirus (HPV) during a 3-year period in women 51 years of age were compared with rates in younger women to provide data on cervical screening for women >50 years of age. Paired, cytologically negative, archived cervical smears taken 3 years apart from 710 women in Nottingham, United Kingdom, were retrieved and tested for HPV infection with polymerase chain reaction (PCR) with GP5+/6+ primers. Seventy-one (21.3%) of 333 women 51 years of age who were HPV negative at baseline were positive 3 years later. This percentage was higher than the corresponding acquisition rates among women 21 (15.2%), 31 (14.1%), and 41 (13.3%) years of age, although these differences were not significant. This retrospective study shows that HPV-negative women >50 years of age can acquire HPV and, therefore, require cervical screening.

Prospective studies of young women (<25 years of age) have reported high rates of cervical human papillomavirus (HPV) acquisition; the average duration of these infections is <1 year (*1**–**4*). Little evidence shows that the converse is true among older women, considering the decline in HPV prevalence that has been found with age (*5**,**6*). In studies from Brazil and Colombia, an incidence of high-risk (carcinogenic) HPV of ≈5% per year was observed among women >35 years of age (*7**,**8*); recent data from Costa Rica showed that the rate of high-risk HPV acquisition during a 5-year interval decreased slightly with increasing age (*9*). All these studies were conducted in areas where the risk of cervical cancer is considered to be high. In a small study from Canada, 7.7% of women 45–49 years of age who were negative for high-risk HPV at baseline tested positive 1 year later, similar to the rate in women 20–25 years of age (*10*). From studies of women in adulthood, estimates of the percentage of HPV infections (high and low risk) that persist >12 months range from 25% to 50% (*10**–**13*); HPV persistence after 5 years increased with age in the study from Costa Rica (*9*).

Data on acquisition and clearance of HPV in women 50–64 years of age in the United Kingdom and other Western nations are needed. The presence of HPV is a requisite to develop invasive cancer of the cervix (*14*). Therefore, women within this age range with cytologically negative specimens, tested with a sensitive testing method such as the polymerase chain reaction (PCR), should have a virtually zero lifetime risk of developing cervical cancer, unless they acquire a new HPV infection. A mathematic modeling study based on the UK screening population estimated that not providing screening for HPV-negative women at age 50 would save as much as 25% of resources for smear tests and 18% for colposcopies; however, the savings would be accompanied by an increase in the incidence of invasive cancers of ≈2/100,000 women annually (*15*). These estimates would be more accurate if the risk of acquiring high-risk HPV infection at these ages was known.

In this study, archived cervical smears were used to compare rates of HPV acquisition during a 3-year period (1 screening interval in the United Kingdom) between women of different ages. This approach also allowed us to estimate rates of HPV persistence and clearance during the same 3-year interval for women whose baseline smears were HPV positive.

## Methods

Anonymous data were provided by the Nottingham Cervical Screening Laboratory, which holds computerized details of all cytology smears of women who have lived in Nottingham since 1987. This laboratory also stores all specimens from women for whom routine cervical screenings were conducted by general practitioners in this area. All specimens used for this study were cytologically normal and had been stored for >10 years (in accordance with UK legal requirements) but were scheduled for routine destruction.

Women eligible for this study were 21, 31, 41, or 51 years of age when a normal baseline smear was taken in 1988. We intended to select a sample of 400 women 51 years of age and 100 women from each of the other age groups to participate in the study. The main entry criterion was a normal cervical smear taken in 1988, followed by a technically adequate subsequent smear (of any cytologic grade) taken from October 1990 to December 1991. The sample for women 51 years of age was supplemented by including women whose baseline smear was obtained in 1989 and who had a subsequent smear taken before July 1992; all eligible women of this age were selected. Women 21, 31, or 41 years of age were selected randomly from the list of participants who met the main entry criteria. For all participants, results from subsequent cytology examinations conducted through December 2002 were provided by the Nottingham Cervical Screening Laboratory, including any cervical biopsy results from which histologic confirmation of cervical intraepithelial neoplasia (CIN, grades 1–3) or cervical cancer was obtained.

When cytology slides were retrieved for HPV testing, all identifiers (name, date of birth, and cytology number) were replaced with a unique study number. Only investigators based at the cytology laboratory (P.V., J.J.) had access to clinical data. These investigators did not have access to HPV testing results for specific patients. Another investigator (M.G.) was responsible for linking HPV test results from the same patient with subsequent cytology results and had access to data on screening histories but not information through which patients could be identified. The study was approved by the Nottingham City Hospital ethics committee.

Cytology slides were immersed in xylene (40 mL) for 2–3 days to remove coverslips. Cells were then scraped with a sterile scalpel blade into an Eppendorf tube containing ethanol (1 mL) and centrifuged to remove any trace of xylene. DNA was extracted by using Qiagen (Crawley, West Sussex, United Kingdom) DNA extraction kits (*16*).

HPV DNA was amplified by using real time PCR with Mx4000 (Stratagene, La Jolla, CA, USA) (*17*). The GP5+ and GP6+ consensus primer pairs in the L1 region of the HPV genome identified infection with any genital HPV type (*18*). The PCR master mix Quantitect (Qiagen) (*19*) contained optimized amounts of SYBR green dye (Molecular Probes, Inc., Eugene, OR, USA) to which primers (5 pmoL/tube) and DNA template (5 μL) were added. Positive controls containing tubes of HPV 16 DNA diluted at various strengths (0.01–10 pg/tube) and negative controls containing the PCR master mix but no DNA template were included in all assays. Forty amplification cycles were performed. Samples that were HPV positive with the GP5+/6+ primer sequence were tested with type-specific HPV16 and HPV18 primers located in the E7 667–686 and 753–774 regions of the genome, respectively (Sequences: HPV16 forward primer, 5´-GAT GAA ATA GAT GGT CCAGC-3´; HPV 16 reverse primer, 5´-GCT TTG TAC GCA CAA CCG AAG C-3´; HPV18 forward primer, 5´-TGA AAT TCC GGT TGA ACC TTC-3´; and HPV 18 reverse primer, 5´GGT CGT CTG CTG AGC TTT CT-3´). A total of 471 cervical DNA samples collected for related studies (although not from women in this study) were tested for the β-globin gene to assess the integrity of the DNA extraction process; 456 (96.8%) tested β-globin positive.

HPV acquisition was calculated as the percentage of women who were HPV negative at baseline but HPV positive on follow-up (and the converse applied for HPV clearance). Clearance of baseline infections was assumed if the equivalent test at follow-up was negative. Nonsymmetric confidence intervals were calculated to measure acquisition and clearance, as these are more appropriate when observed percentages are low (*20*). Rates of HPV acquisition in women of different ages were compared with the χ^2^ test; Fisher exact test was used as a consequence of small numbers for HPV clearance. Rates of HPV prevalence (at baseline and follow-up), acquisition, and clearance across the 4 ages were compared by using the χ^2^ test for trend.

## Results

Cervical smears were retrieved for HPV testing for 710 women (104 were 21 years of age, 105 were 31 years of age, 105 were 41 years of age, and 396 were 51 years of age). Of the study sample (N = 710), 11 were subsequently excluded because of a cervical abnormality before their baseline smear. Follow-up smears could not be retrieved for 27 women; the smear was cytologically abnormal in 20 cases, and the follow-up smear could not be retrieved in the 7 remaining cases. Sixteen women were excluded because an HPV result could not be ascertained at either time because of insufficient DNA. The final sample was 656 women. The mean length of time between the baseline and follow-up smear was 3.08 years (range 1.98–3.84 years). For 76% of the sample (n = 499), the time gap was 2.75–3.25 years. The average time gap between the smears did not vary substantially between age groups.

Rates of HPV infection in this sample are shown in Table 1. In 1988, rates of HPV infection declined with age; the lowest rate was seen in women 51 years of age (10.5%). Three years later, the trend was reversed with the highest rate of HPV in women who were 51 years of age at baseline. A test to determine HPV infection trend with age was significant at baseline and borderline significant at follow-up (Table 1).

**Table 1:** Human papillomavirus (HPV) positivity rates in baseline and follow-up samples

Age (baseline)	Baseline, 1988; no. HPV positive (%)	Follow-up, 1991; no. HPV positive (%)
21 (n = 86)	20 (23.3)	13 (15.1)
31 (n = 98)	13 (13.3)	15 (15.3)
41 (n = 100)	17 (17.0)	14 (14.0)
51 (n = 372)	39 (10.5)	81 (21.8)
	χ^2^ (trend): p = 0.004	χ^2^ (trend): p = 0.059

Age-specific rates of HPV acquisition and clearance (any genital type amplified by the GP5+/6+ primer pair) are shown in Table 2. The HPV acquisition rate was highest for women who were 51 years of age at baseline, although this rate was not significantly higher than the corresponding rate for any of the other ages. A test for trend in the acquisition rates across the 4 ages reached borderline significance (χ^2^ trend = 3.18, p = 0.07). Clearance of HPV infection was lowest for women 51 years of age, although again, no substantial differences were observed between this rate and those for the other 3 ages, and the trend in clearance rates across the 4 ages was not significant (χ^2^ trend = 0.72, p = 0.40). When analyses for overall HPV acquisition and clearance were conducted again and included the 20 women with abnormal cytology at follow-up, assuming that these were HPV positive, the results for both HPV acquisition and clearance remained similar.

**Table 2:** Numbers of women infected with human papillomavirus (HPV) at baseline and follow-up and HPV acquisition and clearance rates by age

Age (baseline)	HPV status – (1988)		HPV status + (1988)		% HPV acquisition in 3 y (95% CI)*	% HPV clearance in 3 y (95% CI)
	– (1991)	+ (1991)	– (1991)	+ (1991)		
21	56	10	17	3	15.2† (8.4–25.7)	85.0‡ (64.0–94.8)
31	73	12	10	3	14.1† (8.3–23.1)	76.9‡ (49.7–91.8)
41	72	11	14	3	13.3† (7.6–22.0)	82.4‡ (59.0–85.4)
51	262	71	29	10	21.3 (17.3–26.0)	74.4 (58.9–85.4)
Total	463	104	70	19	18.3 (15.4–21.7)	78.7 (69.0–85.9)

*CI, confidence interval. †Not significantly different from acquisition rate in women 51 years of age when using χ^2^test (p>0.05). ‡Not significantly different from clearance rate in women 51 years of age when using Fisher exact test (p>0.05).

Type-specific HPV acquisition and clearance rates are shown in Table 3. Twenty (11 in women 51 years of age) new HPV16 infections were found in this sample; 24 (14 in women 51 years of age) new HPV18 infections were found. The low number of new HPV16 and HPV18 infections meant that the confidence intervals for the estimated rates of acquisition of these types were wide. Linear trends to assess rates of acquisition across age were not significant for either HPV16 (χ^2^ trend 0.08, p = 0.77) or HPV18 (χ^2^ trend 0.02, p = 0.89). Three women (21, 31, and 51 years of age, respectively) acquired both HPV16 and HPV18 infections at follow-up, after testing negative for both at baseline. Of the 27 HPV16 or HPV18 infections that were found at baseline over all 4 ages, only 3 were still present at the time of follow-up. This finding indicated overall clearance rates were close to 90% for both HPV16 and HPV18 (Table 3).

**Table 3:** Numbers of women infected with human papillomavirus (HPV) 16 and 18 at baseline and follow-up and HPV16 and HPV18 acquisition and clearance rates by age

Age (y, baseline)	HPV status – (1988)		HPV status + (1988)		% HPV acquisition in 3 y (95% CI)	% HPV clearance in 3 y (95% CI)*
	– (1991)	+ (1991)	– (1991)	+ (1991)		
HPV16						
21	79	3	3	1	3.7 (1.3–10.2)	–
31	94	3	1	0	3.1 (1.1–8.7)	–
41	94	3	3	0	3.1 (1.1–8.7)	–
51	360	11	1	0	3.0 (1.7–5.2)	–
Total	627	20	8	1	3.1 (2.0–4.7)	88.9 (56.5–98.0)
HPV18						
21	77	2	6	1	2.5 (0.7–8.8)	–
31	89	5	3	1	5.3 (2.3–11.9)	–
41	95	3	2	0	3.1 (1.0–8.6)	–
51	352	14	5	0	3.8 (2.3–6.3)	–
Total	613	24	16	2	3.8 (2.5–5.5)	88.9 (67.1–96.9)

*Clearance rates of HPV 16/18 were not calculated for individual ages because of insufficient numbers; CI, confidence interval.

Abnormal findings, including symptoms of mild severity in 10 women and moderate severity in 6 women, developed in 38 women in this study during a follow-up period of 11 years; severe dyskaryosis developed in 5 women. CIN or cancer was histologically confirmed in 8 women; CIN grade 1 was diagnosed in 4 women, CIN 3 was diagnosed in 3, and invasive squamous carcinoma was diagnosed in 1. Cytologic and histologic results stratified by HPV status in 1988 and 1991 are summarized in Table 4. Rates of cervical abnormality during follow-up were slightly higher for women who were HPV positive at baseline, although HPV status at either time was not significantly associated with subsequent abnormal cytologic results (Table 5). Two of the 5 women with severe dyskaryosis, however, had acquired HPV at the time of the second smear (Table 4). Both of these women were 51 years of age at baseline. Squamous carcinoma was diagnosed in 1 of these women in 1998 (7 years after HPV was detected). This woman was negative for HPV types 16 and 18. The other woman also had the cytologic diagnosis in 1998; however, no biopsy result was recorded on this occasion. This woman was positive for both HPV16 and HPV18 at baseline.

**Table 4:** Association between human papillomavirus (HPV) infection in 1988 and 1991 and subsequent cytologic and histologic outcomes

Grade*	HPV status – (1988)		HPV status + (1988)	
	– (1991)	+ (1991)	– (1991)	+ (1991)
Normal	440	97	62	19
Borderline	11	2	4	0
Mild	6	2	2	0
Moderate	3	1	2	0
Severe	3	2	0	0
Any abnormal cytologic results	23	7	8	0

*Worst grade of dyskaryosis diagnosed during 11 years of cytologic follow-up.

**Table 5:** Human papillomavirus (HPV) status and cytologic outcome summarizing results presented in Table 4

HPV status	Cytologic outcome		Total	p*
	Normal, n (%)	Abnormal, n (%)		
1988				
HPV –	537 (94.7)	30 (5.3)	567	0.22
HPV +	81 (91.0)	8 (9.0)	89	
1991				
HPV –	502 (94.2)	31 (5.8)	533	1.0
HPV +	116 (94.3)	7 (5.7)	123	

*Comparing rates of subsequent abnormality between HPV + and – women by using Fisher exact test.

## Discussion

Our study found a high 3-year rate of HPV acquisition rate in women 51 years of age at baseline. Although differences in HPV acquisition with age were not statistically significant, this rate was at least as high as that observed among younger women. This rate is equivalent to the 1-year acquisition rate for high-risk HPV in a Canadian study of a sample of 39 women 45–49 years of age (*10*). Our estimated incidence among 51-year-old women was slightly higher than the rate of high-risk HPV (6.1/100) found in 50- to 54-year-old women in a study from Colombia (*8*), a country with a high rate of cervical cancer. The combined acquisition rate for HPV types 16 and 18 in this study was ≈7%. Only type-specific PCR was conducted for these types, a limitation of this study. Further research is, therefore, required to estimate the combined acquisition rate for all high-risk HPV among older women (and, conversely, to determine whether a substantial proportion of incident infections are with low-risk HPV).

A selection criterion for this study was a cytologically normal cervical smear taken during 1988. In the United Kingdom, changes to the cervical screening program had just been implemented at that time, and coverage rates of the UK screening population were ≈50% (*21*). Whether this change could have caused selection bias remains speculative; women who received cervical screening may have been at higher risk than all eligible women. Another criterion was that all women in the study have cytologically normal results at both phases of the study. As stated earlier, only a small number of women (n = 20) had abnormal cytologic findings at follow-up, and when analyses that indicated these women were HPV positive were conducted again, no noticeable impact was seen on results.

The high rate of HPV acquisition in 51-year-old women may be due to either a higher-than-anticipated rate of new partners among older women or partner infidelity. However, the former supposition is not supported by data from 2 large-scale national surveys of sexual behavior among women in the United Kingdom conducted in 1990 and 2000 (*22**,**23*). The second of these surveys found that only 10.9% of women 35–44 years of age reported a new sex partner in the year preceding the survey, compared with 39.2% of women 16–24 years of age (*23*). Therefore, a 3-year HPV acquisition rate of 21% for women 51 years of age would be higher than expected, based solely on the rate of new partner acquisition among a group of women 35–44 years of age. The 1990 version of this survey, however, reported that 5.4% of men in the 45- to 59-year age group reported >2 sex partners during the previous year (*22*). While these may not have all been new sex partners, and the rate just among married (or cohabiting) men may be lower (separate data not available), this finding indicates that a small part of the total HPV acquisition rate could be a consequence of partner infidelity.

Also, the high rate of HPV acquisition in 51-year-old women may be due to the reemergence of latent HPV infections caused by hormonal changes resulting from menopause or alterations to the cervix caused by hormone replacement therapy. A latent infection can be defined as one where HPV genomes are established in the basal cells but differentiation of the host cells does not take place (*24*). If such infections were below detection with increasingly sensitive PCR technology until the point of reactivation, then women could possibly become HPV positive in the absence of sexual activity. This possibility could also explain the finding of a second age-related peak in HPV prevalence in some South American populations after 55 years of age (*6**,**25**,**26*). However, less evidence for this second peak in HPV prevalence is seen in the United Kingdom and other countries where the risk of cervical cancer is lower.

Additionally, false-negative results occurring during the first phase of testing would cause HPV infections present at baseline and follow-up (i.e., persistent infections) to be misclassified as incident infections, therefore overestimating rates of HPV acquisition. This study relied on archived cervical smears taken a decade earlier to detect HPV infection. While studies have validated the use of this type of specimen by showing agreement between results from archived and fresh specimens from the same women (*27*), concerns exist that the quality of extracted DNA may be poorer for older specimens because of changes in the methods of sampling and fixation (*28*).

A less understood but still plausible reason for these results also relates to the use of archived cervical smears; the possibility of cross-contamination of samples at the time of collection could have accounted for false-positive results at the second phase of testing. This contamination could have occurred either where cervical smears were taken or at the screening laboratory during fixation and staining. In a study from Sweden, this risk of cross-contamination was largely excluded when smears with registration numbers adjacent to the HPV-positive study smears were also tested for HPV (*29*). Results from this study are difficult to generalize to the present setting, where the risk of such cross-contamination could only be assessed if the quality of procedures conducted in doctors' offices and screening laboratories between 1988 and 1991 could be examined.

This report also contains details of HPV infection 3-year clearance rates for women who tested positive in the first phase of the study. As baseline rates of HPV infection in this sample were 10%–25%, depending on age (Table 1), few smears were included for comparison in the analysis of HPV clearance, which resulted in insufficient statistical power for clearance rates between women of different ages to be compared. Among women 51 years of age, a 74% HPV 3-year clearance rate was observed. The potential for false-negative results at the second phase of testing means that these HPV clearance rates are likely overestimated.

In the United Kingdom, screening intervals were recently lengthened to 5 years for women 50–64 years of age, after an analysis of UK screening data showed that the protective effect after a negative cervical smear persisted longer for older women (*30*). Whether cervical screening could be discontinued after 50 years of age for low-risk women has also been debated (*31**–**33*). Whether HPV testing could identify low-risk women would depend on the natural history of the virus in older women and the personal and social implications of introducing a test for a sexually transmitted disease into a national screening program (*34*). Our findings do not support discontinuing cervical screening for HPV-negative women at age 50 because of the high risk of HPV acquisition observed in women 51 years of age during a 3-year interval. By conducting a prospective study in which follow-up is ensured over a period of time at 4- to 6-month intervals, many of the limitations of this study could be circumvented. The probability of viral persistence and progression of incident HPV infections among older women in the United Kingdom and other western countries also should be recognized, as only persistent HPV infections lead to cervical cancer (*35*). Outcome data from this study are limited; further data are required from larger prospective studies to determine more precisely the positive and negative predictive values (for high-grade CIN and cancer) of incident HPV infections that develop in middle age. These issues require consideration before the true impact of discontinuing cervical screening programs worldwide for HPV-negative women >50 years of age can be accurately ascertained.
